# Elevational Variation in Organ Size and Brain–Viscera Energetic Trade-Offs in the Minshan Toad (*Bufo minshanicus*)

**DOI:** 10.3390/biology15161373

**Published:** 2026-08-12

**Authors:** Yaohui Deng, Binwen Liu, Liming Wang, Tonglei Yu

**Affiliations:** 1 College of Life Science, Xinyang Normal University, Xinyang 464000, China; 2 International Joint Research Laboratory for Global Change Ecology, School of Life Sciences, Henan University, Kaifeng 475004, China

**Keywords:** expensive brain hypothesis, elevational gradient, organ trade-off, body condition, *Bufo minshanicus*, energetic constraint

## Abstract

Energy trade-offs underlying brain size evolution represent a central topic in evolutionary biology. The Expensive Brain Hypothesis (EBH) posits that the high energetic costs of large brains must be compensated for by increased energy intake or reduced allocation to other costly tissues and functions. We examined the Minshan toad (*Bufo minshanicus*) along an elevational gradient (2594–3441 m) on the eastern Tibetan Plateau, measuring relative sizes of the brain, five visceral organs, and two reproductive organs, as well as body condition (SMI). Body size and condition significantly decreased with elevation. Relative brain mass showed a non-significant increasing trend with elevation, whereas relative masses of the liver, heart, and alimentary tract significantly increased, and lung mass significantly decreased, with pronounced sexual dimorphism. Relative brain mass was positively correlated with most viscera and male reproductive organs, while a significant brain–ovary trade-off occurred only in females at mid elevation. Overall, elevational environmental conditions shape divergent organ mass covariation patterns. High-altitude habitats may relax energetic constraints to allow coordinated organ development, while moderate mid-altitude stress induces brain–ovary resource trade-offs in females. Our findings do not universally contradict the EBH and instead demonstrate that energetic trade-offs are strongly context-dependent rather than ubiquitous across elevational gradients.

## Simple Summary

Brain energetic trade-offs represent a core unresolved issue in evolutionary biology. We surveyed Minshan toads across an elevational gradient on the eastern Tibetan Plateau to test the Expensive Brain Hypothesis. Body size and physical condition declined at higher altitudes. Relative brain mass trended slightly upward, while the liver, heart, and digestive tract grew larger, and lungs shrank; clear sexual differences in organs were observed. The brain positively correlated with most visceral organs and male testes, yet only mid-altitude females showed a brain–ovary energy trade-off. High-elevation habitats may present milder energetic constraints, which could correlate with coordinated organ enlargement in wild populations. Our results do not universally contradict the Expensive Brain Hypothesis, as organ trade-offs depend on environmental stress at different elevations.

## 1. Introduction

Variation in brain size and its adaptive significance in animals has long attracted research attention from evolutionary biologists and neuroscientists [1]. Enlarged brains can boost cognitive abilities amid diverse social and environmental pressures, potentially elevating individual fitness when species inhabit harsh or newly emerged habitats [1]. Nevertheless, the brain is one of the most energetically expensive organs in the body, requiring a continuous resource supply to sustain physiological function [2,3]. Consequently, investment in a large brain often increases individual metabolic costs, which can lead to reduced investment in other tissues and functions (e.g., somatic maintenance and reproductive output [4,5]).

Aiello and Wheeler first proposed a trade-off between brain size and gut size in primates, termed the expensive tissue hypothesis [6]. Two related yet distinct energetic frameworks underpin vertebrate brain evolution research: the ETH and the Expensive Brain Hypothesis (EBH). The original ETH is narrow in scope and only predicts a brain–digestive tract trade-off specific to primates. In contrast, Isler and van Schaik [5] developed the generalized EBH to expand this original idea beyond gut tissue. Under the EBH, elevated brain metabolic costs can be offset via two pathways: increased total energy intake or reduced resource allocation to any metabolically costly tissues and life-history traits (e.g., heart, liver, gonads, somatic growth, and fat reserves). This study adopts the broader EBH framework to interpret context-dependent brain–organ covariation across elevational populations. Fundamentally, energetic trade-offs are shaped by genetic covariance among phenotypic traits [7]. The EBH further predicts positive correlations between brain size and basal metabolic rate (BMR), whereas larger brains tend to trade off against energetically pricey somatic tissues and life-history characteristics governing somatic growth and reproductive investment [5,8].

Most empirical tests of brain evolution focus on endothermic mammals and birds [6,9,10,11,12,13,14,15]. Recent work has shifted toward ectothermic vertebrates, particularly amphibians and fish [16,17,18]. Notably, the strongest empirical support for the expensive tissue hypothesis (ETH) comes not from homeotherms but from ectotherms, including fishes and anurans. Most mammalian and avian studies have failed to detect clear negative brain–gut correlations, instead reporting trade-offs between brain size and life-history traits [5,8,9]. In contrast, consistent negative associations between brain and digestive tract size have been documented across amphibians and in Lake Tanganyika cichlids [17,18]. Negative relationships between brain size and visceral fat storage have also been reported in pipefish and anurans [19,20]. Experimental evidence from guppies further supports a brain–gut trade-off [16], although several studies in other fishes and amphibians have not detected trade-offs between brain size and other metabolically costly organs [21,22,23,24]. Ectotherms may therefore be particularly suitable for testing energetic trade-off hypotheses. Brain evolution in anurans is also tightly linked to anti-predator adaptations such as camouflage [25], and domestication can lead to brain reduction [26]. Despite possessing similarly costly neural tissue [27], ectotherms exhibit substantially lower whole-body metabolic rates [28] and less-temperature-dependent brain metabolism [29].

The Minshan toad (*Bufo minshanicus*) is widely distributed across a broad elevational gradient (1700–3700 m a.s.l.), although breeding populations below 2600 m are rare (Table 1 and Table 2 [30]). Such extensive elevational variation exposes populations to divergent selective pressures [31] and provides an excellent system to test the EBH. *Bufo minshanicus* is a large terrestrial anuran inhabiting high-elevation croplands, marshes, meadows, and forest edges across the eastern Tibetan Plateau [30]. Terrestrial species may be particularly sensitive to environmental extremes compared with aquatic taxa due to prolonged exposure to ambient conditions [32,33]. High-elevation environments impose strong energetic constraints via hypoxia, low temperatures, short annual activity periods, and high climatic variability [34,35]. Theoretical frameworks predict that under such resource limitations, vertebrates may offset the metabolic expense of large brains by reallocating energy away from other energetically demanding organs. However, considerable interindividual variation in resource acquisition suggests energetic constraints may not always be strong enough to produce measurable trade-offs [36,37]. Nevertheless, elevational gradients provide a powerful framework for understanding evolutionary processes and fitness consequences associated with organ size variation [38,39,40].

In this study, we examined variation in brain size, five metabolically costly visceral organs (heart, liver, alimentary tract, kidneys, and lungs), and two reproductive organs in both sexes of *B. minshanicus* from three sites along an elevational gradient. We also quantified energy storage and tested potential trade-offs between brain size and other costly organs, including reproductive tissues. Prior investigations have explored elevational variation in brain size in closely related toad species [41]. We addressed three questions:

(1) How do brain size, visceral organs, and reproductive organs vary along elevational gradients?
(2) Does brain size exhibit trade-offs with visceral and/or reproductive organs?
(3) Does elevational variation in energy storage influence such trade-offs?

Nutrient metabolism and cardiac function are key adaptive mechanisms for this toad in high-elevation environments [42,43]. Accordingly, we put forward three testable predictions: (1) relative brain mass would decline with rising elevation, while metabolically costly visceral organs would expand under severe energy limitation; (2) negative energetic trade-offs would exist between brain tissue and visceral organs; (3) these trade-offs may become more pronounced at high elevations driven by scarce, fluctuating food supplies.

## 2. Materials and Methods

### 2.1. Study Site and Sample Collection

We sampled three populations of sexually mature *B. minshanicus* across an elevational gradient of 2594–3441 m on the eastern Tibetan Plateau (Table 1 and Table 2). Sampling occurred during the breeding season (late March to mid-April), which is delayed at higher elevations, ensuring that the individuals were sexually mature [31]. For each population, we obtained the annual total precipitation (ATP) from WorldClim v2.1 (https://worldclim.org/, (accessed on 10 December 2025), [44]) and the normalized difference vegetation index (NDVI) from the Resource and Environmental Science Data Center (https://www.resdc.cn, (accessed on 10 December 2025), [45]). These data characterized climatic and resource conditions and facilitated the interpretation of organ size variation and energetic trade-offs [12].

Adult *B. minshanicus* populations were collected by hand from breeding habitats. Sex was identified in situ—males by nuptial pads and mating calls; females by a distended abdomen, indicating mature oocytes. Toads were housed individually in opaque plastic containers (diameter: 16.75 cm) with 2 cm of fresh water at ambient temperature. We measured snout–vent length (SVL) with vernier calipers (Shanghai Precision Scientific Instrument Co., Ltd., Shanghai, China; precision: 0.1 mm) and body mass with an electronic balance (Ohaus Instrument (Shanghai) Co., Ltd., Shanghai, China; precision: 0.01 g). We used the scaled mass index (SMI; [46]) as an index of body condition and energy reserves, a reliable metric widely applied in amphibian research [47]. SMI was calculated as SMI = M_i_ × (L_0_/L_i_)^b_SMA_, where *Mi* and *L_i_* were body mass and SVL of individuals, respectively, while *L*_0_ was an arbitrary value of *L* (e.g., here, the mean value within each population); *b_SMA_* was the SMA regression coefficient for natural-log converted body mass versus snout–vent length (SVL), calculated using lmodel2 package [48]. We chose SMI instead of raw SVL or body mass as the covariate. SVL only reflects skeletal length, while crude body mass is easily affected by temporary hydration and feeding status. SMI integrates length and mass via allometric correction to reliably reflect long-term individual energy reserves, which is essential for unbiasedly testing brain–organ energetic trade-offs across elevations and sexes.

To ensure full hydration, individuals were acclimated for 48 h in fresh water before euthanasia. Euthanasia was performed by immersion in a 300 mg/L solution of tricaine methane sulfonate (TMS; Sigma-Aldrich, St. Louis, MO, USA) and confirmed by pithing. Carcasses were fixed in 4% buffered formalin. The extended fixation period was necessitated by logistical constraints associated with sampling in remote, high-altitude environments, where immediate processing of a large number of individuals was not feasible. Fixation enabled standardized dissection and measurement under controlled laboratory conditions. Following six months of fixation, the brain and major organs (heart, lungs, kidneys, liver, alimentary tract, testes, and ovaries) were dissected and weighed to the nearest 0.1 mg using an electronic balance. Sex and maturity were confirmed. This approach is validated by previous studies demonstrating that formalin preservation time does not significantly affect brain or other organ masses in frogs [18,49].

### 2.2. Statistical Analysis

We conducted comprehensive pre-model diagnostics for ordinary least squares (OLS) regression prior to all analyses. After log_10_ transformation of morphometric variables and *Z*-score standardization of continuous predictors, Kolmogorov–Smirnov normality tests yielded *p* < 0.05 for nearly all response variables, and Levene’s homoscedasticity tests also produced *p* < 0.05 across elevation and sex subgroups. These results clearly indicate significant departures from residual normality and homogeneous variance (the standard threshold for satisfying regression assumptions is *p* > 0.05). Residual diagnostic plots further confirmed this violation, and multiple influential outliers were also detected in organ mass and body size datasets.

Conventional OLS regression is highly sensitive to non-normal residuals, heteroscedasticity, and extreme outliers. Under such severe assumption violations, OLS coefficient estimates, standard errors, and significance *p*-values will be systematically biased, and the resulting outputs are statistically unreliable and cannot serve as valid comparative references. For this reason, we did not implement a parallel OLS sensitivity analysis. Instead, robust MM-estimation regression was uniformly adopted across all models, as this framework downweighs the impact of outliers and relaxes strict residual distribution requirements to produce stable, trustworthy parameter estimates.

We first examined relationships among SVL, SMI, and elevation using robust regression. SMI and SVL were dependent variables; sex and elevation were independent variables. Sex was included because strong sexual size dimorphism occurs in this species [31]. Analyses used the robustbase package (v0.99-7).

We then assessed relationships between relative organ size and elevation using robust regression. Log-transformed organ sizes (brain, heart, liver, alimentary tract, kidneys, lungs, testes, and ovaries) were dependent variables. Sex and elevation were independent variables, and log-transformed SMI was included as a covariate to account for body size and energy storage.

We used robust MM-estimation regression to test relationships between brain size and other organs across elevations. After controlling for SMI, elevation, and sex (only SMI and elevation for reproductive organs), we evaluated associations between brain mass and each organ and tested whether elevation moderated these relationships. MM estimation uses Tukey’s biweight criterion to reduce bias from outliers. Inferences were based on HC3 heteroscedasticity-robust standard errors. Continuous predictors were *Z*-score-standardized prior to analysis to avoid multicollinearity. Variance inflation factors (VIF < 4) confirmed no severe collinearity. We then applied robust MM-estimation separately at each elevation to examine brain–organ relationships while controlling for SMI and sex (only SMI and elevation for reproductive organs). Reproductive organs were analyzed separately by sex to avoid confounding sexual dimorphism.

We also used robust regression to examine pairwise relationships among all visceral and reproductive organs while controlling for SMI, sex, and elevation. An organ × elevation interaction term was included to test whether elevation modified pairwise relationships. All organ variables were standardized, and VIF values confirmed no multicollinearity. Separate models were fitted for each pair, and residual diagnostics validated model fit.

To visualize multivariate phenotypic differentiation across sexes and three elevational groups, we further conducted principal component analysis (PCA) on all nine log-transformed morphological traits (SMI, SVL, bmass, heart, liver, kidneys, gut, eggs, and sperm). All variables were *Z*-score-standardized before PCA to eliminate unit differences. We extracted the first two principal components (PC1 and PC2) for two-dimensional ordination and calculated variable loadings to interpret trait contributions to phenotypic variation. PCA scatter plots with 95% confidence ellipses were generated to display group clustering patterns.

All analyses were performed in R version 4.5.2 (R Core Team, 2025; software homepage: https://www.R-project.org/, accessed on 25 February 2026) [50]). Data processing used the tidyverse package (v2.0.0) [51]. Robust regression analyses used the robustbase package. Allometric correction was performed using GroupStruct v0.1.0 [52]. SMI was calculated using lmodel2 (v1.7-3) [48]. Missing data were handled using the naniar package (v0.6.1) [53]; missingness was randomly distributed (*p* > 0.05). All figures were generated using the ggplot2 package (v3.4.4) [54]. Residual normality and homoscedasticity were verified for all models. All PCA computations were implemented via base R prcomp and ggplot2 packages.

## 3. Results

### 3.1. SVL, SMI, and Organ Size Changes Along Elevational Gradients

**Figure 1:**
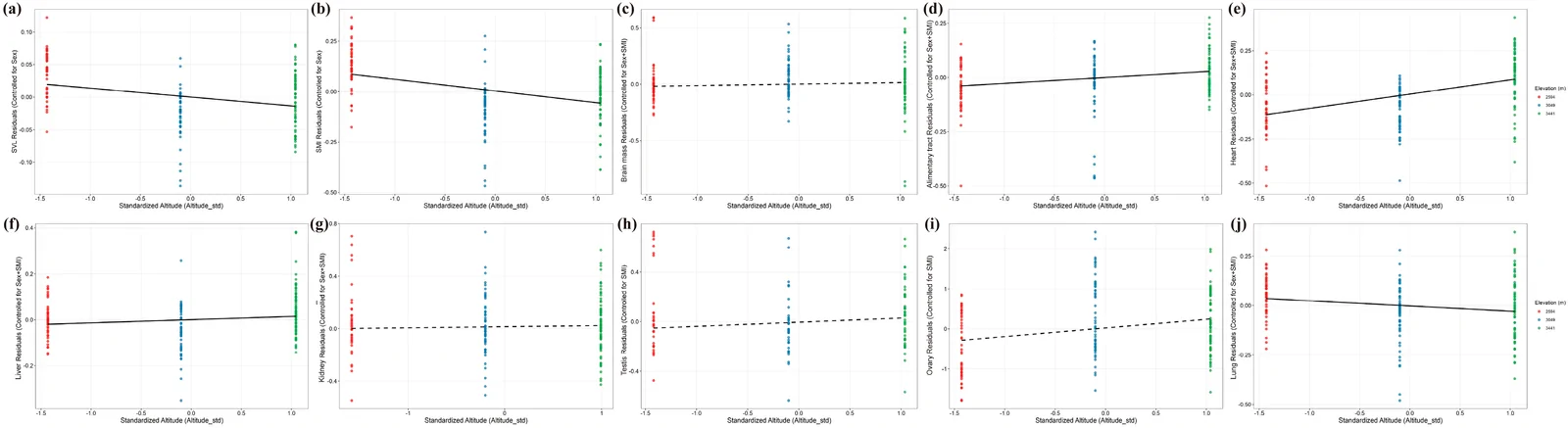
Variation in body size, body condition, and relative organ mass of *Bufo minshanicus* along an elevational gradient. (**a**,**b**) Snout–vent length (SVL) and scaled mass index (SMI); (**c**) relative brain mass; (**d**–**j**) relative mass of alimentary tract, heart, liver, kidneys, testes, ovaries, and lungs, respectively. Dashed lines represent non-significant correlations. Elevation was Z-score-standardized to mitigate multicollinearity. All fitted lines are derived from robust MM-estimation regression.

Both SVL and SMI significantly decreased with elevation (Table 3). Robust regression showed significant negative correlations between SVL and elevation (*t* = −3.987, *p* < 0.001; Figure 1a) and between SMI and elevation (*t* = −5.795, *p* < 0.001; Figure 1b). Toads at high elevations had smaller SVL and SMI than those at low elevations. Significant sexual dimorphism occurred in SVL and SMI; males had smaller values than females (Table 3).

Relative brain mass exhibited a statistically non-significant positive trend with elevation (Table 3; Figure 1c) after accounting for SMI (*t* = 3.288, *p* = 0.001) and sex (*t* = 0.369, *p* = 0.712). Sexual dimorphism in relative brain size was not significant (Table 3).

Relative alimentary tract, heart, and liver masses were significantly and positively associated with elevation after controlling for SMI (all *p* < 0.032; Figure 1d–f) and sex (all *p* < 0.001). Relative kidney, testis, and ovary masses showed positive but non-significant trends (all *p* > 0.054; Table 3; Figure 1g–i). In contrast, relative lung mass significantly decreased with elevation (*t* = −2.572, *p* = 0.011; Figure 1j). Males had significantly smaller relative alimentary tract mass (*p* < 0.001) and marginally smaller kidney mass (*p* = 0.102), but significantly larger relative liver, heart, and lung masses than females (all *p* < 0.012; Table 3).

### 3.2. Co-Variation Between Brain Mass and Other Visceral Organs

**Figure 2:**
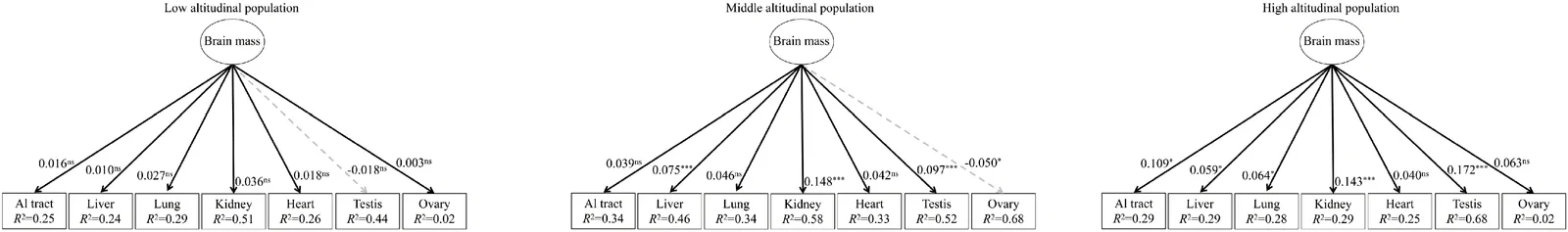
Elevational differences in the correlations between relative brain mass and visceral/reproductive organ mass of *Bufo minshanicus*. All fitted lines represent robust regression predictions. * *p* < 0.05, *** *p* < 0.001, ns = non-significant.

Relative brain mass (adjusted for SMI) was significantly and positively correlated with alimentary tract, liver, lung, kidney, and testis masses: alimentary tract (*β* = 0.052 ± 0.018, *t* = 2.817, *p* = 0.005), liver (*β* = 0.044 ± 0.014, *t* = 3.015, *p* = 0.003), kidneys (*β* = 0.128 ± 0.022, *t* = 5.762, *p* < 0.001), lungs (*β* = 0.037 ± 0.019, *t* = 1.981, *p* = 0.049), and testes (*β* = 0.109 ± 0.019, *t* = 5.757, *p* < 0.001; Table 4, Figure 2). Elevation showed no significant interactive effects with alimentary tract, liver, lungs, or kidneys (all *p* > 0.087). Only the testis × elevation interaction was significant (*β* = 0.073 ± 0.016, *t* = 4.424, *p* < 0.001).

**Figure 3:**
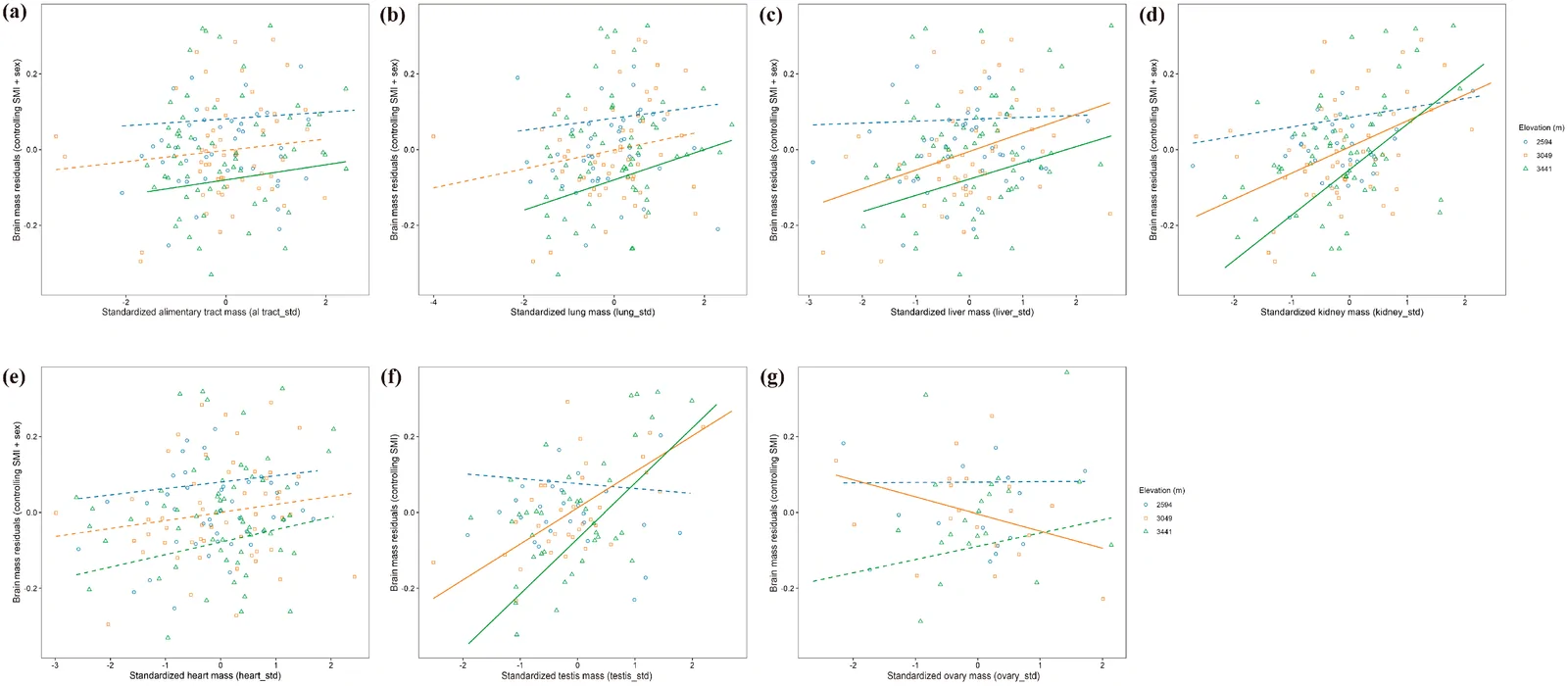
Relationships between relative brain mass and the mass of visceral and reproductive organs in *Bufo minshanicus* across elevations. (**a**–**f**) Correlations between brain mass and alimentary tract, lungs, liver, kidneys, heart, and testes; (**g**) correlation between brain mass and ovary mass. A significant negative brain–ovary trade-off was observed at mid elevation. Dashed lines represent non-significant correlations. All fitted lines are generated via robust MM-estimation regression. Note: Slight vertical offsets (+0.08 for 2594 m, −0.08 for 3441 m) were applied to trend lines of different elevations to improve readability. The slopes of all trend lines remain consistent with the results of robust regression analyses.

Stratified analyses revealed strong elevational variation in brain–organ relationships. The alimentary tract and lungs were significantly and positively correlated with relative brain mass only at high elevation (alimentary tract: *t* = 2.231, *p* = 0.029; lungs: *t* = 2.411, *p* = 0.019; Figure 3a,b), with no significant relationships at low or mid elevation (both *p* > 0.05; Figure 2). Liver and kidney masses were significantly positively correlated with relative brain mass at mid and high elevations (both *p* < 0.001 at mid elevation; *p* < 0.05 at high elevation; Figure 3c,d), but not at low elevation (both *p* > 0.10; Figure 2). Similarly, testis mass showed a non-significant negative correlation with brain mass at low elevation (*t* = −0.441, *p* = 0.663; Figure 2), but significant positive correlations at mid and high elevations (both *p* < 0.001, Figure 3f). These correlational patterns suggest that high-elevation conditions may reinforce coordinated enlargement of the brain and other somatic organs.

In contrast, relative brain mass showed a non-significant positive association with heart mass (*β* = 0.027 ± 0.016, *t* = 1.744, *p* = 0.083; Figure 3e). No significant brain–heart relationships occurred within any elevation zone (all *p* > 0.107; Figure 2), and no heart × elevation interaction was detected (*t* = 0.942, *p* > 0.05). Heart mass had little independent effect on brain mass, and elevation did not modulate this relationship.

For ovaries, neither brain mass nor the ovary × elevation interaction was significant (both *p* > 0.246). However, stratified analyses revealed a significant negative correlation between ovary mass and brain mass at mid elevation (*t* = −2.214, *p* = 0.041; Figure 2 and Figure 3g), with non-significant positive correlations at low and high elevations (both *p* > 0.395). This pattern indicates a context-dependent energetic trade-off between reproductive investment (ovaries) and neural investment (brain) under moderate environmental stress.

### 3.3. Co-Variation Among Organ Masses

**Figure 4:**
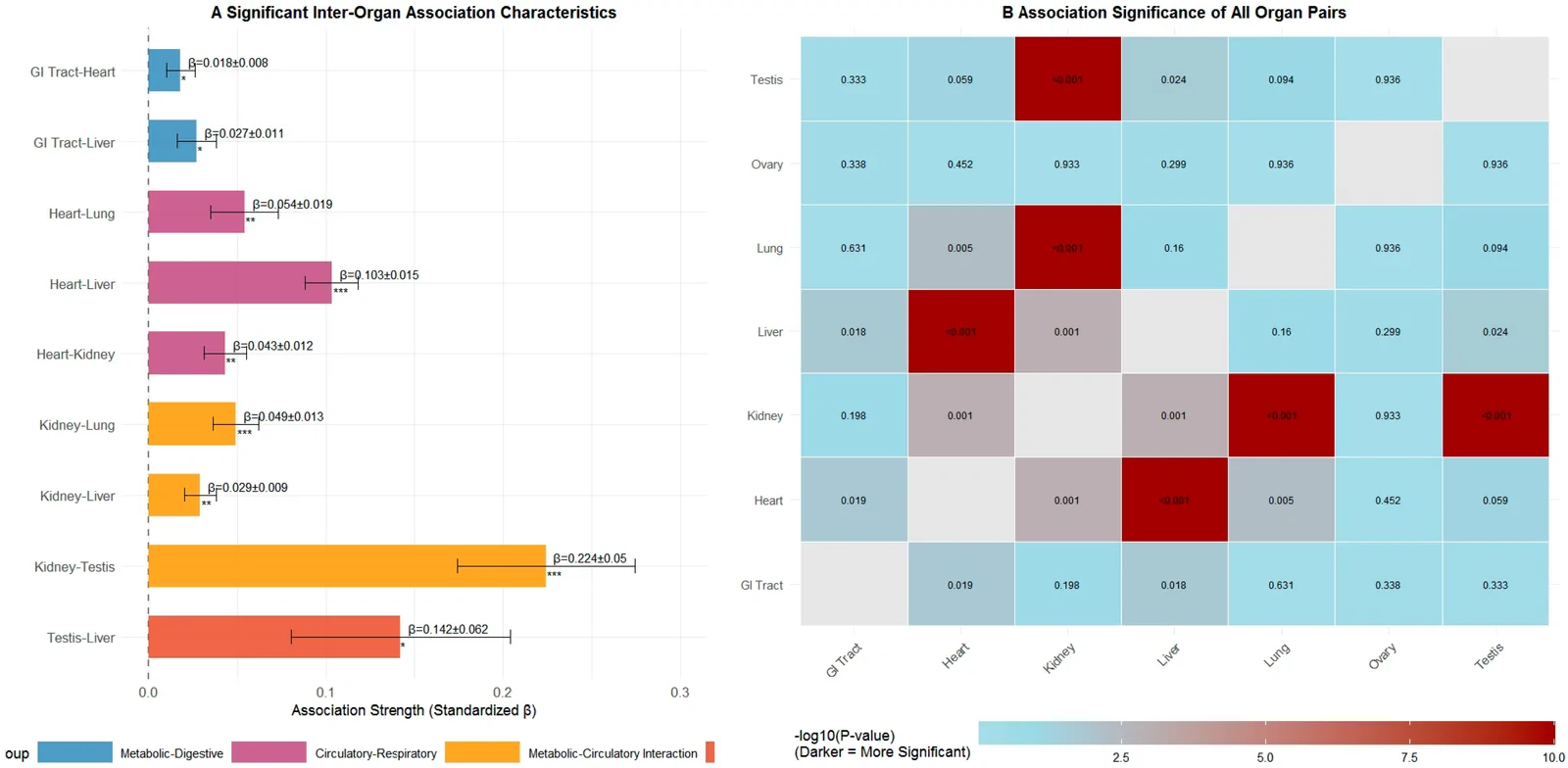
Pairwise correlations among visceral and reproductive organ masses in *Bufo minshanicus*. All fitted regression lines are based on robust MM-estimation models. * *p* < 0.05, ** *p* < 0.01, *** *p* < 0.001.

Multiple significant positive correlations were detected among organ masses (all *p* < 0.05; Figure 4). Specifically, the alimentary tract was positively correlated with the heart (*β* = 0.018 ± 0.008, *t* = 2.364, *p* = 0.019) and liver (*β* = 0.027 ± 0.011, *t* = 2.389, *p* = 0.018). The heart was positively correlated with the lungs (*β* = 0.054 ± 0.019, *t* = 2.864, *p* = 0.005), liver (*β* = 0.103 ± 0.015, *t* = 6.755, *p* < 0.001), and kidneys (*β* = 0.043 ± 0.012, *t* = 3.519, *p* = 0.001). The kidneys were positively correlated with the liver (*β* = 0.029 ± 0.009, *t* = 3.306, *p* = 0.001), lungs (*β* = 0.049 ± 0.013, *t* = 3.681, *p* < 0.001), and testes (*β* = 0.224 ± 0.050, *t* = 4.449, *p* < 0.001). The testes were also positively correlated with the liver (*β* = 0.142 ± 0.062, *t* = 2.299, *p* = 0.024; Figure 4).

For the moderating effect of elevation, only the lung–elevation interaction was significant (*β* = −0.020 ± 0.009, *t* = −2.121, *p* = 0.035). All other organ pair × elevation interactions were non-significant (all *p* > 0.111), showing stable inter-organ coordination except lung tissue. Remaining pairwise organ correlations were weak and non-significant.

### 3.4. Multivariate Phenotypic Differentiation via Principal Component Analysis

**Figure 5:**
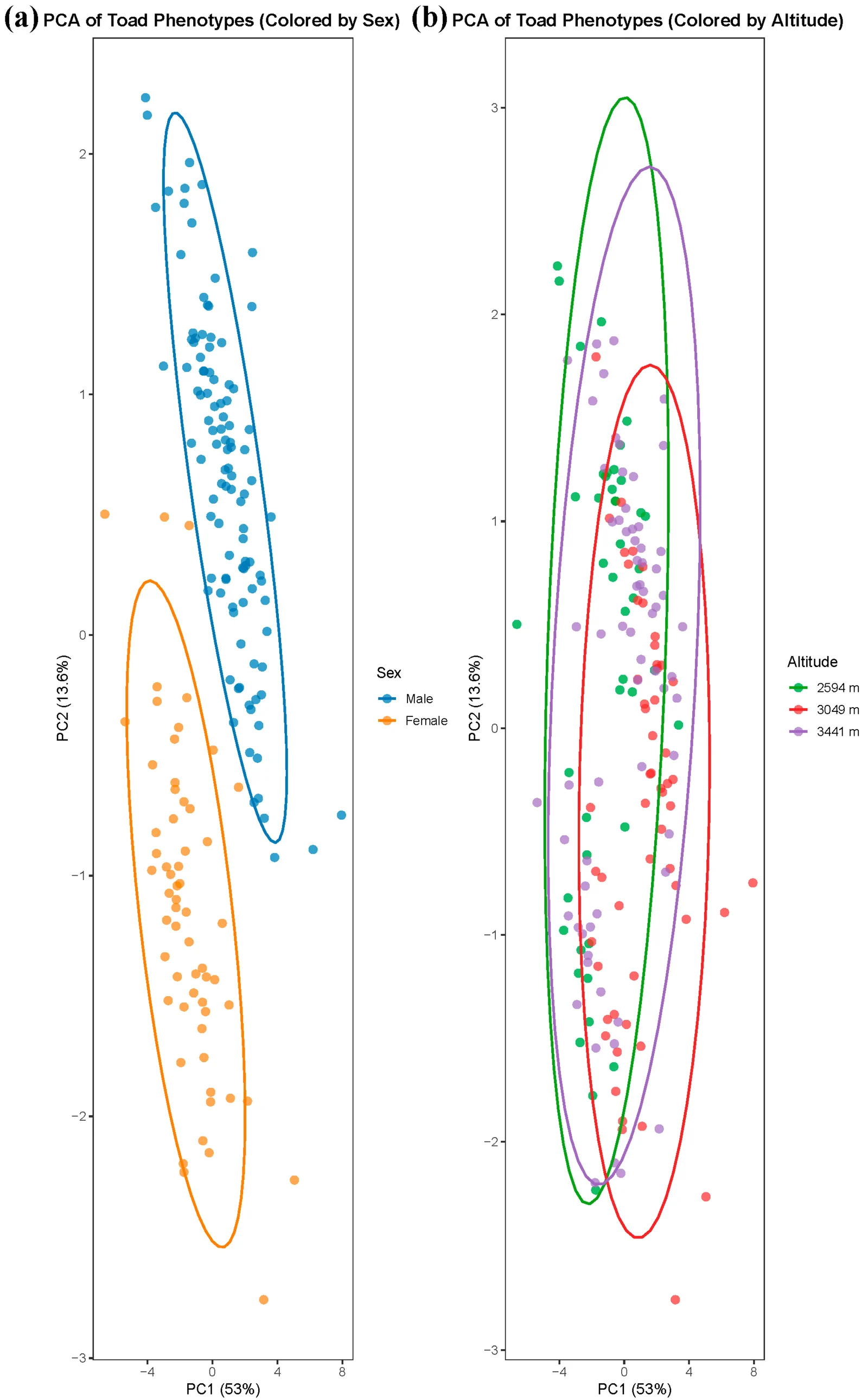
Two-panel PCA ordination plot showing multivariate phenotypic clustering of *Bufo minshanicus*. (**a**) Samples colored by sex; (**b**) samples colored by elevation. Shaded areas = 95% confidence ellipses. Axis labels show explained variance percentages.

PCA was conducted on nine log-transformed, standardized morphological traits (Figure 5). PC1 and PC2 together explained 66.6% of the total phenotypic variance (53.0% and 13.6%, respectively). Every variable had negative PC1 loadings; SMI (−0.403) and SVL (−0.391) contributed most to this body-size composite axis. PC2 separated reproductive tissue (gonad loading = −0.635) from metabolic viscera (liver = 0.481, heart = 0.336).

Sex groups formed fully non-overlapping elliptical clusters; males distributed at higher PC1 coordinates. Three elevation populations partially overlapped but exhibited ordered PC1 shifts from low (2594 m) to high altitude (3441 m).

## 4. Discussion

We analyzed elevational visceral organ mass variation in wild Minshan toads. Given the lack of common-garden and genetic tests, observed organ clines cannot be definitively attributed to evolutionary adaptation, as phenotypic plasticity and transient environmental effects remain confounding factors. We detected clear elevational shifts in relative organ mass; the liver, heart, and alimentary tract expanded at higher elevations, lung mass decreased, and all organs exhibited marked sexual dimorphism. These morphological shifts likely constitute coordinated physiological responses to highland cold, hypoxia, and resource limitation, though direct metabolic and prey availability data are missing to validate this mechanism. Our results partially agree with Yao et al. [55] but diverge from Li et al. [56] and Zhao et al. [57]. Yao et al. [55] observed reduced brain size and enlarged visceral organs alongside brain–viscera trade-offs in high-altitude *B. gargarizans.* Li et al. [56] showed declining dry visceral mass in lab-raised conspecifics, implying metabolic suppression as a highland coping strategy. Zhao et al. [57] found no elevational organ enlargement in *B. andrewsi*, demonstrating that oxygen and energy demand do not universally shape organ size across toad species. These inter-study discrepancies reveal organ size evolution is jointly shaped by taxon-specific energy allocation, energetic trade-offs, and context-dependent fitness outcomes [58,59].

SVL and SMI significantly decreased with elevation. Relative brain mass tended to increase with elevation, while most visceral and reproductive organs increased in relative size, except lung mass. A significant trade-off between brain size and ovarian investment occurred only at mid elevation. Most other organs showed significant positive correlations with brain mass, and strong positive covariation occurred among most visceral and reproductive organs. Our results do not completely negate the EBH; brain–gonad trade-offs only appear under moderate mid-altitude stress, while high elevations have relaxed energy constraints enabling simultaneous organ growth. This context-dependent pattern proves EBH predictions are not universal along elevation gradients, providing new evidence for debates on EBH applicability in amphibians and reptiles [60].

### 4.1. Elevational Phenotypic Variation in Visceral Organ Mass

High-altitude stressors likely drive organ morphological divergence, though our analysis relies solely on static morphological data without direct physiological validation. Consistent with multivariate PCA ordination demonstrating uphill shifts in integrated organ size across elevational populations, univariate robust regressions revealed significant elevational increases in relative liver, heart, and alimentary tract mass, alongside reduced lung mass. This suite of coordinated organ shifts may represent altered resource allocation to metabolic and digestive tissues. However, we lack direct field data on prey availability to validate their adaptive functional consequences. Larger livers could facilitate cold tolerance through enhanced energy storage and glycolipid turnover, a pattern consistent with documented energy storage strategies in high-altitude amphibians [58,59,61,62]. Cold environments alter energy allocation in *Bufo* species, causing suppressed feeding and retarded somatic growth [62,63,64]. Congeneric toads show inconsistent hepatic and intestinal elevational clines [55,56,57], and such plasticity varies widely across ectotherms under lineage-specific energy allocation rules [65,66]. Relative heart mass increased significantly with elevation. This morphological shift could stem from plastic physiological adjustment to hypoxic conditions, rather than heritable evolutionary adaptation. Larger cardiac tissue may elevate cardiac output and systemic blood flow, potentially improving oxygen transport across body tissues. This finding matches the positive heart-size–elevation association documented in the spot-legged treefrog *Polypedates megacephalus* [49]. Likewise, high-altitude deer mice (*Peromyscus maniculatus*) have evolved enlarged cardiac tissue by suppressing harmful phenotypic plasticity, consistent with the organ-expansion strategy observed in anurans [38]. Transcriptomic data confirm cardiac tissue is core to highland adaptation in *B. gargarizans* [42]. In contrast, *N. parkeri* has evolved smaller hearts due to divergent energy priorities; *B. gargarizans* trades brain tissue for visceral function, while *N. parkeri* prioritizes reproduction [55,67].

High-altitude toads exhibited longer digestive tracts, a trait that may correlate with low-quality dietary resources. Low temperatures and limited prey abundance can suppress digestive enzyme activity in ectotherms; longer intestinal tracts might prolong food retention time and partially compensate for reduced nutrient uptake efficiency. In line with this rule, omnivorous cyprinid *Capoeta banarescui* has developed longer intestines at high elevations to cope with an increasingly herbivorous diet [68]. Still, this elevational pattern is not universal across ectotherms; *P. megacephalus* has the largest gut at mid elevation, while *B. spinulosus* and highland lizards have reduced gut length to allocate energy to fat storage [49,69,70,71]. These divergent yet functionally equivalent morphological shifts prove that food abundance and dietary composition dominate digestive-tract adaptive evolution across highland ectotherms.

Relative testis and ovary masses displayed non-significant positive elevational trends, shaped by combined physiological and environmental constraints. High elevations shorten breeding windows. Though individuals tend to boost reproductive investment to maximize single-clutch output, large intraspecific variation and fluctuating ambient temperatures obscure this trend statistically, consistent with enhanced reproductive input under cold conditions in the plateau brown frog *R. kukunoris* [72] and the general highland anuran strategy of safeguarding reproductive allocation. Kidney mass had a non-significant upward trend; anuran aquaporin-mediated cutaneous water retention relieves selection pressure for renal enlargement under highland aridity and hypoxia [6,73].

Relative lung mass decreased markedly with increasing elevation. This clinal pattern could signal a shift toward cutaneous respiration and modified tissue energy partitioning at high altitude, though microstructural lung traits (e.g., alveolar density) remain uncharacterized in our dataset [55]. Underlying microstructural optimization (e.g., alveolar density) awaits future histological validation. Arboreal *P. megacephalus* increases lung volume uphill for aerial respiration, whereas terrestrial anurans like our study species downsize lungs and depend more on efficient skin gas exchange in cool humid highlands [49]. We tentatively infer that energetic limitation, breeding strategy, and habitat features may together shape elevational organ variation; this integrated interpretation remains untested due to absent field resource surveys.

### 4.2. Inter- and Intraspecific Inconsistencies in Organ Size Plasticity and Context-Dependent Energetic Trade-Offs

Adaptive organ differentiation along elevational gradients varies markedly across species and conspecific populations. Our results revealed elevated relative heart mass at high elevations, consistent with cardiac enlargement in highland *P. megacephalus* and *B. gargarizans* [49,55]. In contrast, highland *N. parkeri* develops smaller hearts [67]. Such divergence stems from disparate energy allocation priorities; highland *B. gargarizans* sacrifices brain investment to sustain essential visceral organs including the heart, forming significant brain–viscera trade-offs [55], whereas *N. parkeri* prioritizes reproductive development over cardiac growth [67]. Both *Rana chensinensis* and *B. minshanicus* exhibit shortened annual active and foraging durations at higher latitudes or elevations [58]. However, the two species adopt divergent energy allocation strategies in response to elevational stress. *Rana chensinensis* prioritizes metabolic organs such as the liver while reducing investment in carcass tissues under combined constraints of short activity time and severe food shortage [58]. In contrast, high-elevation populations of *B. minshanicus* may gain access to sufficient food resources that mitigate the fitness costs of shortened annual foraging windows. If prey availability is comparable across elevations, energetic constraints would be relaxed at high altitudes, permitting concurrent enlargement of metabolic and reproductive organs. Moderate environmental pressure at mid elevations, by contrast, may generate competitive resource allocation between costly tissues. We note, however, that this mechanistic interpretation cannot be confirmed without quantitative surveys of local prey density.

Intraspecific divergence also exists within *B. gargarizans*, while no significant elevation-related organ shifts were detected in its close relative *B. andrewsi* [57,74,75]. Li et al. [56] reported declining visceral organ mass along elevational gradients, whereas Yao et al. [55] and our study found positive elevational increases in the liver, alimentary tract, and kidneys alongside brain–viscera trade-offs at high elevations. These discrepancies mainly originate from differences in sampled elevation ranges and corresponding energetic constraints; Li et al. [56] surveyed mid–low elevations (1000–3000 m) with mild resource limitation, whereas Yao et al. ([55]; 785–3239 m) and the present study covered broad high-elevation gradients where energy scarcity is pronounced, as reflected by declining scaled mass index values [55]. Widespread anurans exhibit high phenotypic plasticity, and local adaptation can produce divergent resource allocation strategies among geographically separated populations [74]. Similar adaptations in brain structure have been observed in other mammals across environmental gradients [39,76].

We only detected a statistically significant negative correlation between brain and ovarian mass in mid-elevation females. This observation implies that tissue resource allocation trade-offs may depend heavily on elevation-specific environmental stress regimes, though causal relationships cannot be established from our cross-sectional sampling. Precipitation increases with elevation across our sampling zone; high-altitude alpine grasslands contrast with lowland agricultural habitats. Though high elevations have cooler temperatures, prey availability remains comparable to lowlands. Yak grazing in the Zoige Wetlands sustains abundant Aphodiidae beetles, a key food resource for highland anurans including *B. minshanicus* and *R. kukunoris* [77]; sparse alpine vegetation also widens visual range and enhances foraging success. Combined, these factors relieve energetic constraints at high elevations, allowing simultaneous investment in metabolic and reproductive organs without detectable trade-offs. Mid elevation creates moderate environmental stress; cooler temperatures reduce insect prey, and stronger predation limits active foraging time. Mild resource limitation creates allocation conflicts in females, generating the brain–ovary trade-off predicted by the EBH [6,10,55]. This conditional trade-off matches the elevation-dependent brain-reproductive trade-offs reported in *B. gargarizans* [55]. Our mid-elevation sampling range overlaps the high-altitude gradient surveyed by Yao et al. [55], suggesting that this elevation band forms a critical stress threshold triggering brain–gonad resource competition in *Bufo* toads.

### 4.3. Sexual Dimorphism in Organ Size

Sexual divergence in relative organ mass could originate from sex-differentiated reproductive energy budgets and microhabitat use. All evolutionary interpretations regarding this dimorphism remain tentative, however, as we did not quantify mating energy costs or individual female fecundity in this study. All three elevational populations showed stable female-biased sexual size dimorphism; females had larger SVL and SMI than males at every altitude, and body size and condition declined with elevation in both sexes while dimorphism magnitude remained unchanged. This female-larger SSD is broadly conserved across the *Bufo* species complex [63,64,78,79].

Males displayed enlarged relative livers, hearts, and lungs, traits that may support energetically costly breeding behaviors such as advertisement calling and male–male competition. Females, by contrast, invested proportionally more tissue mass into ovaries, coinciding with smaller relative digestive and renal organs. This sex-biased allocation matches energy budget patterns in *R. kukunoris* and *B. gargarizans* [55,62,65,72], as well as other *Bufo* species [63,64,78,79,80]. Females need stronger digestive capacity to accumulate nutrients for egg development, while males prioritize metabolic tissue over digestive function for breeding activities [81,82]. However, we detected no sexual divergence in relative brain mass in *B. minshanicus*, implying sex-dependent brain investment is not conserved across anuran taxa but evolves under lineage-specific high-elevation environmental pressures.

## 5. Conclusions

We examined elevational organ variation and brain–viscera energetic trade-offs in highland *Bufo minshanicus* spanning 2594–3441 m. Elevation reduced body size, body condition, and relative lung mass, yet enlarged the heart, liver, and digestive tract; organ allocation exhibited consistent sexual dimorphism, with males prioritizing metabolic and respiratory tissues for breeding and females investing more in digestion and ovaries, while relative brain mass weakly increased at high elevations without sex divergence. Consistent positive covariation between the brain and most visceral organs as well as male testes at mid and high elevations indicates coordinated organ growth under relaxed energetic limitations, whereas a marked brain–ovary trade-off only occurs in mid-elevation females exposed to moderate environmental stress; together, these findings demonstrate that EBH-predicted trade-offs are context-dependent instead of universal. Broad positive correlations across metabolically vital viscera reflected adaptive physiological coordination to high-altitude hypoxia and resource scarcity, and PCA verified elevation and sex as primary drivers of integrated phenotypic differentiation. Collectively, our correlational data suggest that highland energetic pressures may modulate tissue resource partitioning across elevations. Moderate environmental limitation at mid altitudes could trigger competitive allocation between neural and reproductive tissues in females, while comparatively abundant resources at high elevations might eliminate such resource conflicts and permit coordinated growth of metabolically expensive organs. This study supplies novel intraspecific amphibian evidence for conditional EBH responses on plateaus, and advocates future common-garden and molecular assays to separate plastic and genetic sources of elevational organ clines.

## 6. Limitations

The present study has several key limitations. This field observational study only identifies correlational links between elevation and organ mass. Controlled common-garden manipulations will be necessary to disentangle the independent effects of high-altitude environmental stress, inter-population genetic divergence, and short-term phenotypic plasticity. Accordingly, all evolutionary and energetic inferences presented in this observational study should be treated as unvalidated hypotheses. Sampling was restricted to the breeding season, precluding the analysis of seasonal shifts in organ resource allocation. We also lack in situ measurements of prey density, individual metabolism, energy intake and lipid storage across elevations, which limit mechanistic explanations for elevation-dependent brain–organ energetic trade-offs predicted by the EBH. No physiological functional tests or tissue transcriptomic profiling were conducted to characterize organ plasticity, despite transcriptomic adaptation to high altitude being documented in closely related toad species (Chen et al. [83]). Future research should adopt denser elevational sampling, larger sample sizes, and integrated morphological, physiological, and molecular analyses to disentangle the multiple drivers shaping elevational organ size clines.

## Figures and Tables

**Table 1:** Sampling location, sample size, body size, and organ mass data for male *Bufo minshanicus* populations at three elevations on the eastern Tibetan Plateau.

Populations	Altitude (m)	Latitude	Brain Mass (g)	SVL (mm)	SMI	Heart (mg)	Liver (mg)	Kidney (mg)	Gut (mg)	Lung (mg)	Gonad (mg)
Shiyazhuang (SYZ)	2594	36.68	0.14 ± 0.019 (29)	68.89 ± 1.11 (29)	61.80 ± 3.16 (29)	574.10 ± 39.44 (29)	1590.80 ± 78.54 (29)	454.70 ± 74.67 (24)	1010.30 ± 40.26 (29)	814.90 ± 42.92 (29)	250.50 ± 44.34 (29)
Guoguori (GGR)	3441	34.49	0.11 ± 0.008 (35)	60.53 ± 0.80 (35)	38.90 ± 2.12 (35)	384.10 ± 23.72 (35)	954.60 ± 50.64 (35)	242.00 ± 18.94 (35)	993.20 ± 40.70 (35)	466.80 ± 33.93 (35)	126.80 ± 16.25 (34)
Gongquhu (GQH)	3049	34.29	0.12 ± 0.010 (43)	63.21 ± 1.02 (43)	41.24 ± 1.75 (43)	636.00 ± 38.20 (43)	1323.40 ± 63.61 (43)	307.20 ± 26.99 (43)	1066.50 ± 36.84 (43)	480.70 ± 24.76 (43)	164.00 ± 18.36 (43)

Notes: Values in parentheses represent total individuals sampled for the corresponding organ. Unequal sample sizes across organs within a single population arose because minor tissues (kidneys and gonads) were damaged or lost during formalin fixation and dissection after six months of long-term field preservation. SVL = snout–vent length; SMI = somatic mass index; gonad represents testes in males and ovaries in females. SYZ, GQH, and GGR are abbreviations for Shiyazhuang, Gongquhu, and Guoguori, respectively.

**Table 2:** Sampling location, sample size, body size, and organ mass data for female *Bufo minshanicus* populations at three elevations on the eastern Tibetan Plateau.

Populations	Altitude (m)	Latitude	Brain Mass (g)	SVL (mm)	SMI	Heart (mg)	Liver (mg)	Kidney (mg)	Gut (mg)	Lung (mg)	Gonad (mg)
Shiyazhuang (SYZ)	2594	36.68	0.17 ± 0.035 (16)	80.53 ± 1.35 (16)	91.61 ± 4.96 (16)	504.20 ± 42.73 (16)	1486.60 ± 88.16 (16)	577.00 ± 167.43 (11)	1678.40 ± 122.91 (16)	1019.70 ± 87.29 (16)	20,122.20 ± 1495.02 (16)
Guoguori (GGR)	3441	34.49	0.15 ± 0.015 (20)	67.88 ± 1.27 (20)	50.24 ± 2.81 (20)	420.50 ± 26.99 (20)	936.00 ± 62.78 (20)	476.50 ± 59.49 (20)	1439.30 ± 112.42 (20)	445.00 ± 46.52 (20)	12,575.50 ± 1022.05 (20)
Gongquhu (GQH)	3049	34.29	0.15 ± 0.018 (24)	74.81 ± 1.23 (24)	69.02 ± 3.53 (24)	808.50 ± 48.07 (24)	1231.40 ± 49.04 (24)	446.60 ± 50.11 (24)	2018.50 ± 122.96 (24)	735.30 ± 74.82 (24)	18,391.10 ± 930.26 (24)

Notes: Values in parentheses represent total individuals sampled for the corresponding organ. Unequal sample sizes across organs within a single population arose because minor tissues (kidneys and gonads) were damaged or lost during formalin fixation and dissection after six months of long-term field preservation. SVL = snout–vent length; SMI = somatic mass index; gonad represents testes in males and ovaries in females. SYZ, GQH, and GGR are abbreviations for Shiyazhuang, Gongquhu, and Guoguori, respectively.

**Table 3:** Core statistical indicators of robust regression analyses for each phenotypic trait in *Bufo minshanicus*.

Dependent Variable	Independent Variable	Regression Coefficient	SE	t	*p*
SVL	Sex	0.06731	0.00687	9.798	<0.001
	Altitude	−0.00004	0.00001	−3.987	<0.001
SMI	Sex	0.18167	0.02169	8.374	<0.001
	Altitude	−0.00017	0.00003	−5.795	<0.001
Brain Mass	Sex	0.01824	0.04940	0.369	0.712
	SMI	0.47868	0.14558	3.288	0.001
	Altitude	0.00005	0.00005	0.963	0.337
Gut	Sex	0.16128	0.02496	16.461	<0.001
	SMI	0.36609	0.08901	4.113	<0.001
	Altitude	0.00010	0.00003	3.641	<0.001
Liver	Sex	−0.15507	0.02037	−7.613	<0.001
	SMI	0.81711	0.07071	11.555	<0.001
	Altitude	0.00005	0.00002	2.167	0.032
Heart	Sex	−0.07782	0.02932	−2.654	0.009
	SMI	0.74821	0.09231	8.106	<0.001
	Altitude	0.00028	0.00004	6.866	<0.001
Lung	Sex	−0.07268	0.02845	−2.555	0.012
	SMI	0.86454	0.10104	8.556	<0.001
	Altitude	−0.00009	0.00004	−2.572	0.011
Kidney	Sex	0.04687	0.02845	1.647	0.102
	SMI	0.66480	0.10104	6.580	<0.001
	Altitude	0.00004	0.00004	1.146	0.253
Testis	SMI	0.74157	0.21621	3.430	0.001
	Altitude	0.00014	0.00011	1.328	0.187
Ovary	SMI	0.88347	0.13079	6.755	<0.001
	Altitude	0.00008	0.00004	1.971	0.054

Notes: SE, standard error; SVL, snout–vent length; SMI, somatic mass index.

**Table 4:** Robust regression results for the associations between brain mass and visceral organ traits in *Bufo minshanicus*.

Organ	Independent Variable	VIF (Standardized)	Regression Coefficient	SE	t	*p*
Gut	Sex	1.617	−0.0316	0.036	−0.882	0.379
	SMI	2.441	0.0565	0.018	3.174	0.002
	Altitude	1.459	0.0005	0.014	0.038	0.970
	Gut	2.529	0.0516	0.018	2.817	0.005
	Gut × Altitude	1.032	0.0098	0.012	0.829	0.409
Heart	Sex	1.442	0.0326	0.034	0.947	0.345
	SMI	2.204	0.0651	0.020	3.274	0.001
	Altitude	1.604	0.0044	0.015	0.288	0.774
	Heart	1.552	0.0268	0.015	1.744	0.083
	Heart × Altitude	1.145	0.0010	0.013	0.073	0.942
Liver	Sex	1.900	0.0484	0.031	1.536	0.127
	SMI	3.303	0.0536	0.019	2.855	0.005
	Altitude	1.482	0.0048	0.013	0.357	0.722
	Liver	2.225	0.0435	0.014	3.015	0.003
	Liver × Altitude	1.371	0.0265	0.015	1.721	0.087
Lung	Sex	1.477	0.0212	0.033	0.632	0.528
	SMI	2.891	0.0580	0.022	2.591	0.010
	Altitude	1.436	0.0113	0.015	0.763	0.446
	Lung	2.215	0.0374	0.019	1.981	0.049
	Lung × Altitude	1.331	0.0265	0.015	1.811	0.072
Kidney	Sex	1.365	0.0019	0.028	0.068	0.946
	SMI	1.699	0.0325	0.016	2.093	0.038
	Altitude	1.167	0.0045	0.013	0.344	0.732
	Kidney	1.375	0.1281	0.022	5.762	<0.001
	Kidney × Altitude	1.106	0.0373	0.030	1.257	0.211
Testis	SMI	1.395	0.0343	0.017	2.014	0.047
	Altitude	1.305	0.0037	0.015	0.237	0.813
	Testis	1.203	0.1094	0.019	5.757	<0.001
	Testis × Altitude	1.114	0.0728	0.016	4.424	<0.001
Ovary	SMI	2.082	0.1131	0.096	1.176	0.245
	Altitude	1.558	0.0308	0.026	1.180	0.243
	Ovary	2.039	−0.0575	0.049	−1.172	0.246
	Ovary × Altitude	1.443	0.0032	0.026	0.120	0.905

Variance inflation factor (VIF) < 5 indicates no severe multicollinearity; model reliability is ensured.

## Data Availability

Data have been made available at https://doi.org/10.5281/zenodo.20838967.

## References

[1] Fristoe T.S., Iwaniuk A.N., Botero C.A. (2017). Big brains stabilize populations and facilitate colonization of variable habitats in birds. Nat. Ecol. Evol..

[2] Olesen S.P. (1986). Rapid increase in blood-brain barrier permeability during severe hypoxia and metabolic inhibition. Brain Res..

[3] Singer M.A. (2007). Comparative Physiology, Natural Animal Models and Clinical Medicine: Insights into Clinical Medicine from Animal Adaptations.

[4] Barrickman N.L., Lin M.J. (2010). Encephalization, expensive tissues, and energetics: An examination of the relative costs of brain size in strepsirrhines. Am. J. Phys. Anthropol..

[5] Isler K., van Schaik C.P. (2009). The expensive brain: A framework for explaining evolutionary changes in brain size. J. Hum. Evol..

[6] Aiello L.C., Wheeler P. (1995). The expensive-tissue hypothesis: The brain and the digestive system in human and primate evolution. Curr. Anthropol..

[7] Houle D. (1991). Genetic covariance of fitness correlates: What genetic correlations are made of and why it matters. Evolution.

[8] Heldstab S.A., van Schaik C.P., Isler K. (2022). The economics of brain size evolution in vertebrates. Biol. Rev..

[9] Jones K.E., MacLarnon A.M. (2004). Affording larger brains: Testing hypotheses of mammalian brain evolution on bats. Am. Nat..

[10] Isler K., van Schaik C.P. (2006). Costs of encephalization: The energy trade-off hypothesis tested on birds. J. Hum. Evol..

[11] Pitnick S., Jones K.E., Wilkinson G.S. (2006). Mating system and brain size in bats. Proc. R. Soc. B Biol. Sci..

[12] Navarrete A., van Schaik C.P., Isler K. (2011). Energetics and the evolution of human brain size. Nature.

[13] Jin L., Jiang Y., Han L.X., Luan X.F., Liu X., Liao W.B. (2025). Big-brained alien birds tend to exhibit climatic niche shifts through enhanced behavioral innovation. Integr. Zool..

[14] Li S.B., Liu Y.X., Du X.L., Li G.P., Liao W.B. (2024). Nest complexity correlates with larger brain size but smaller body mass across bird species. Integr. Zool..

[15] Zhong M.J., Wang J., Feng J., Lin A.Q. (2026). Energy constraints and ecological adaptation drive divergent evolution of sensory and cognitive brain regions in bats. Integr. Zool..

[16] Kotrschal A., Rogell B., Bundsen A., Svensson B., Zajitschek S., Brännström I., Immler S., Maklakov A.A., Kolm N. (2013). Artificial selection on relative brain size in the guppy reveals costs and benefits of evolving a larger brain. Curr. Biol..

[17] Tsuboi M., Shoji J., Sogabe A., Ahnesjö I., Kolm N. (2015). Comparative support for the expensive tissue hypothesis: Big brains are correlated with smaller gut and greater parental investment in Lake Tanganyika cichlids. Evolution.

[18] Liao W.B., Lou S.L., Zeng Y., Kotrschal A. (2016). Large brains, small guts: The expensive tissue hypothesis supported within anurans. Am. Nat..

[19] Tsuboi M., Shoji J., Sogabe A., Ahnesjö I., Kolm N. (2016). Within species support for the expensive tissue hypothesis: A negative association between brain size and visceral fat storage in females of the Pacific seaweed pipefish. Ecol. Evol..

[20] Huang Y., Mai C.L., Liao W.B., Kotrschal A. (2020). Body mass variation is negatively associated with brain size: Evidence for the fat-brain trade-off in anurans. Evolution.

[21] Dornburg A., Warren D.L., Zapfe K.L., Morris R., Iglesias T.L., Lamb A., Hogue G., Lukas L., Wong R. (2018). Testing ontogenetic patterns of sexual size dimorphism against expectations of the expensive tissue hypothesis, an intraspecific example using oyster toadfish (*Opsanus tau*). Ecol. Evol..

[22] Mai C.L., Liao J., Zhao L., Liu S.M., Liao W.B. (2017). Brain size evolution in the frog *Fejervarya limnocharis* supports neither the cognitive buffer nor the expensive brain hypothesis. J. Zool..

[23] Warren D.L., Iglesias T.L. (2012). No evidence for the ‘expensive-tissue hypothesis’ from an intraspecific study in a highly variable species. J. Evol. Biol..

[24] Yang S.N., Feng H., Jin L., Zhou Z.M., Liao W.B. (2018). No evidence for the expensive-tissue hypothesis in *Fejervarya limnocharis*. Anim. Biol..

[25] Liao W.B., Jiang Y., Li D.Y., Jin L., Zhong M.J., Qi Y., Lüpold S., Kotrschal A. (2022). Cognition contra camouflage: How the brain mediates predator-driven crypsis evolution. Sci. Adv..

[26] Ning Z.Y., Cao L.S., Wu Y.M., Sun Y.B., Jin L., Liao W.B. (2026). Brain reduction in domestic frog species. Evol. Biol..

[27] Mink J.W., Blumenschine R.J., Adams D.B. (1981). Ratio of central nervous system to body metabolism in vertebrates: Its constancy and functional basis. Am. J. Physiol..

[28] White C.R., Seymour R.S. (2006). The scaling and temperature dependence of vertebrate metabolism. Biol. Lett..

[29] Hochachka P.W., Somero G.N., Hoar W.S., Randall D.J. (1971). Biochemical adaptation to the environment. Fish Physiology.

[30] Fei L., Ye C.Y., Jiang J.P. (2012). Colored Atlas of Chinese Amphibians and Their Distributions.

[31] Yu T.L., Wang D.L., Busam M., Deng Y.H. (2019). Altitudinal variation in body size in *Bufo minshanicus* supports Bergmann’s rule. Evol. Ecol..

[32] Sunday J.M., Bates A.E., Dulvy N.K. (2012). Thermal tolerance and the global redistribution of animals. Nat. Clim. Change.

[33] Hoffmann E.P., Cavanough K.L., Mitchell N.J. (2021). Low desiccation and thermal tolerance constrains a terrestrial amphibian to a rare and disappearing microclimate niche. Conserv. Physiol..

[34] Jiang A., Zhong M.J., Xie M., Lou S.L., Jin L., Robert J., Liao W.B. (2015). Seasonality and age is positively related to brain size in Andrew’s Toad (*Bufo andrewsi*). Evol. Biol..

[35] Muir A.P., Biek R., Thomas R., Mable B.K. (2014). Local adaptation with high gene flow: Temperature parameters drive adaptation to altitude in the common frog (*Rana temporaria*). Mol. Ecol..

[36] Agrawal A.A. (2020). A scale-dependent framework for trade-offs, syndromes, and specialization in organismal biology. Ecology.

[37] Garland T. (2014). Trade-offs. Curr. Biol..

[38] Luquet É., Léna J.P., Miaud C., Plénet S. (2015). Phenotypic divergence of the common toad (*Bufo bufo*) along an altitudinal gradient: Evidence for local adaptation. Heredity.

[39] Velotta J.P., Ivy C.M., Wolf C.J., Scott G.R., Cheviron Z.A. (2018). Maladaptive phenotypic plasticity in cardiac muscle growth is suppressed in high-altitude deer mice. Evolution.

[40] Nengovhela A., Ivy C.M., Scott G.R., Denys C., Taylor P.J. (2023). Counter-gradient variation and the expensive tissue hypothesis explain parallel brain size reductions at high elevation in cricetid and murid rodents. Sci. Rep..

[41] Yao Z., Huang K., Qi Y., Fu J. (2021). Brain size variation along altitudinal gradients in the Asiatic Toad (*Bufo gargarizans*). Ecol. Evol..

[42] Yang W., Qi Y., Lu B., Qiao L., Wu Y., Fu J. (2017). Gene expression variations in high-altitude adaptation: A case study of the Asiatic toad (*Bufo gargarizans*). BMC Genet..

[43] Jiao M., Zhang Y., Liu C., Cao Z., Storey K.B., Niu Y. (2025). Ecometabolomics reveal physiological adaptations of Asiatic toads (*Bufo gargarizans* Cantor, 1842) to different environments along an altitudinal gradient. Front. Zool..

[44] Fick S.E., Hijmans R.J. (2017). WorldClim 2: New 1-km spatial resolution climate surfaces for global land areas. Int. J. Climatol..

[45] Chen T., Feng Z., Zhao H., Wu K. (2020). Dataset of ecosystem services in Beijing and its surrounding areas. Data Brief.

[46] Peig J., Green A.J. (2009). New perspectives for estimating body condition from mass/length data: The scaled mass index as an alternative method. Oikos.

[47] MacCracken J.G., Stebbings J.L. (2012). Test of a body condition index with Amphibians. J. Herpetol..

[48] Legendre P. (2024). lmodel2: Model II Regression.

[49] Zhong M.J., Wang X.Y., Huang Y.Y., Liao W.B. (2017). Altitudinal variation in organ size in *Polypedates megacephalus*. Herpetol. J..

[50] R Core Team (2025). R: A Language and Environment for Statistical Computing.

[51] Wickham H., Averick M., Bryan J., Chang W., McGowan L.D.A., François R., Grolemund G., Hayes A., Henry L., Hester J. (2019). Welcome to the tidyverse. J. Open Source Softw..

[52] Chan K.O., Grismer L.L. (2022). GroupStruct: An R package for allometric size correction. Zootaxa.

[53] Tierney N., Cook D., McBain M., Fay C., O’Hara-Wild M., Hester J., Smith L., Heiss A. (2024). naniar: Data Structures, Summaries, and Visualisations for Missing Data.

[54] Wickham H. (2016). ggplot2: Elegant Graphics for Data Analysis.

[55] Yao Z., Huang K., Qi Y., Fu J. (2025). Does brain size of Asiatic toads (*Bufo gargarizans*) trade-off with other energetically expensive organs along altitudinal gradients?. Evolution.

[56] Li P., Tan S., Yao Z.Y., Fu J.Z., Chen J.F. (2020). Comparisons of heart size of *Bufo gargarizans* from different altitudinal gradients in a common garden environment: Is Hesse’s rule applicable to ectotherms?. Sichuan J. Zool..

[57] Zhao L., Mai C.L., Liu G.H., Liao W.B. (2019). Altitudinal implications in organ size in the Andrew’s toad (*Bufo andrewsi*). Anim. Biol..

[58] Chen W., Zhang L.X., Lu X. (2011). Higher pre-hibernation energy storage in anurans from cold environments: A case study on a temperate frog *Rana chensinensis* along a broad latitudinal and altitudinal gradients. Ann. Zool. Fenn..

[59] Cheng X.F., Shi W.H., Li H.Y., Yu T.L. (2023). Interpopulation variation in prebreeding energy reserves of plateau brown frog (*Rana kukunoris*). Acta Herpetol..

[60] Song Z., Griesser M., Schuppli C., van Schaik C.P. (2023). Does the expensive brain hypothesis apply to amphibians and reptiles?. BMC Ecol. Evol..

[61] Lu X., Li B., Li Y., Ma X., Fellers G.M. (2008). Pre-hibernation energy reserves in a temperate anuran, *Rana chensinensis*, along a relatively fine elevational gradient. Herpetol. J..

[62] Chen W., Wang X., Fan X. (2013). Do anurans living in higher altitudes have higher prehibernation energy storage? Investigations from a high-altitude frog. Herpetol. J..

[63] Tan S., Li P., Yao Z., Liu G., Yue B., Fu J., Chen J. (2021). Metabolic cold adaptation in the Asiatic toad: Intraspecific comparison along an altitudinal gradient. J. Comp. Physiol. B.

[64] Kalayci T.E., Gül S., Dursun C., Karaoğlu H., Özdemir N. (2019). Age structure and body size variation in common toad (*Bufo bufo*, Linnaeus 1758) from three different altitudes in Turkey. Russ. J. Ecol..

[65] Dursun C., Özdemir N., Gül S., Üzüm N., Kutrup B. (2022). Intra-and interspecific elevational variation in keratinized head spines in two closely related *Bufo* species. S. Am. J. Herpetol..

[66] Jönsson K.I., Herczeg G., O’Hara R.B., Söderman F., Ter Schure A.F., Larsson P., Merilä J. (2009). Sexual patterns of prebreeding energy reserves in the common frog *Rana temporaria* along a latitudinal gradient. Ecography.

[67] Hou D., Jia T., Ren Y., Zhu W., Liu P. (2023). Phenotypic trait variations in the frog *Nanorana parkeri*: Differing adaptive strategies to altitude between sexes. J. Vertebr. Biol..

[68] Akin S., Turan H., Kaymak N. (2016). Does diet variation determine the digestive tract length of *Capoeta banarescui* Turan, Kottelat, Ekmekci and Imamoglu, 2006? *J*. Appl. Ichthyol..

[69] Naya D.E., Veloso C., Bozinovic F. (2009). Gut size variation among *Bufo spinulosus* populations along an altitudinal (and dietary) gradient. Ann. Zool. Fenn..

[70] Naya D.E., Bozinovic F. (2009). Digestive plasticity in Andean lizards: Effects of altitude and season on gut size and function. Physiol. Biochem. Zool..

[71] Naya D.E., Veloso C., Bozinovic F. (2008). Physiological flexibility in the Andean lizard *Liolaemus bellii*: Seasonal changes in energy acquisition, storage and expenditure. J. Comp. Physiol. B.

[72] Chen W., Guan T., Ren L., He D., Wang Y., Lu X. (2015). Prehibernation energy storage in Heilongjiang brown frogs (*Rana amurensis*) from five populations in North China. Asian Herpetol. Res..

[73] Suzuki M., Hasegawa T., Ogushi Y., Tanaka S. (2007). Amphibian aquaporins and adaptation to terrestrial environments: A review. Comp. Biochem. Physiol. Part A Mol. Integr. Physiol..

[74] Jiang Y., Luan X.F., Liao W.B. (2023). Anuran brain size predicts food availability-driven population density. Sci. China Life Sci..

[75] Jiang Y., Yuan L.J., Liao W.B. (2024). Chemical defense mediates population density-driven anuran brain size evolution. Sci. China Life Sci..

[76] Taylor P.J., Nengovhela A., Denys C., Scott G.R., Ivy C.M. (2024). Adaptation in brain structure and respiratory and olfactory structures across environmental gradients in African and North American muroid rodents. Integr. Zool..

[77] Zhang J.D., Li Y.J., Dai Q., Wang Y.Z. (2010). Effects of grazing on diet of anurans in Zoige Wetlands, Sichuan, China. Chin. J. Appl. Environ. Biol..

[78] Liao W., Jiang Y., Jin L., Lüpold S. (2023). How hibernation in frogs drives brain and reproductive evolution in opposite directions. eLife.

[79] Dursun C., Özdemir N., Gül S. (2023). Sexual shape dimorphism in *Bufo verrucosissimus* (Pallas, 1814) from Lake Borçka Karagöl, Türkiye. Acta Zool. Acad. Sci. Hung..

[80] Dursun C., Gül S., Özdemir N. (2022). Sexual size and shape dimorphism in Turkish common toads (*Bufo bufo* Linnaeus 1758). Anat. Rec..

[81] Liao W.B., Ma D.L., Jiang A., Cao L.S., Wu H. (2025). Males with greater mating success during male-male competition have larger brain size in the Andrews toad (*Bufo andrewsi*). Asian Herpetol. Res..

[82] Wu H., Su C.Y., Cao L.S., Liao W.B. (2026). Sex recognition and mate choice are positively correlated with relative brain size in male *Bufo andrewsi*. Integr. Zool..

[83] Chen W., Tang Z.H., Fan X.G., Wang Y., Pike D.A. (2013). Maternal investment increases with altitude in a frog on the Tibetan Plateau. J. Evol. Biol..

